# The impact of biological age of red blood cell on *in vitro* endothelial activation markers

**DOI:** 10.3389/fphys.2023.1127103

**Published:** 2023-03-08

**Authors:** Abdulrahman Alshalani, Boukje M. Beuger, Anita M. Tuip-de Boer, Robin van Bruggen, Jason P. Acker, Nicole P. Juffermans

**Affiliations:** ^1^ Chair of Medical and Molecular Genetics Research, Department of Clinical Laboratory Sciences, College of Applied Medical Sciences, King Saud University, Riyadh, Saudi Arabia; ^2^ Laboratory of Experimental Intensive Care and Anesthesiology, University of Amsterdam, Amsterdam, Netherlands; ^3^ Department of Molecular Hematology, Sanquin Research and Landsteiner Laboratory, Academic Medical Center, University of Amsterdam, Amsterdam, Netherlands; ^4^ Department of Laboratory Medicine and Pathology, University of Alberta, Edmonton, AB, Canada; ^5^ Innovation and Portfolio Management, Canadian Blood Services, Edmonton, AB, Canada; ^6^ Department of Intensive Care, Erasmus Medical Center, Rotterdam, Netherlands

**Keywords:** young red blood cell, blood donor, RBC subpopulations, Percoll fractionation, endothelial activation

## Abstract

**Introduction:** Blood donor characteristics influence red blood cell transfusion outcomes. As donor sex affects the distribution of young to old RBCs in the circulation, we hypothesized that the amount of circulating young RBCs in the blood product are associated with immune suppression.

**Materials and Methods:** Blood samples were collected from healthy volunteers and density fractionated into young and old subpopulations. In an activated endothelial cell model, RBC adhesion to endothelium and secretion of endothelial activation markers were assessed. The impact of RBC biological age was also assessed in a T cell proliferation assay and in a whole blood stimulation assay.

**Results:** After Percoll fractionation, young RBCs contained more reticulocytes compared to old RBCs. Young RBCs associated with lower levels of E-selectin, ICAM-1, and vWF from activated endothelial cells compared to old RBCs. RBC subpopulations did not affect T cell proliferation or cytokine responses following whole blood stimulation.

**Conclusion:** Young RBCs contain more reticulocytes which are associated with lower levels of endothelial activation markers compared to old RBCs.

## Introduction

The potential harm of red blood cell transfusion is a highly controversial subject and has been recognized as one of the most frequently stated problems within the field of transfusion medicine. Recently, there is a growing interest in the influence of donor characteristics on the quality of RBC products (Jordan et al., 2016; Kanias et al., 2016; Tzounakas et al., 2016; Kanias et al., 2017; Alshalani et al., 2019) and on post-transfusion patient outcomes (Middelburg et al., 2011; Chasse et al., 2016a; Chasse et al., 2016b; Bjursten et al., 2016; Zeller et al., 2019). Findings of observational studies have varied, but there is a concern towards an association between receiving RBC units from female donors and the risk of mortality (Middelburg et al., 2011; Chasse et al., 2016a; Caram-Deelder et al., 2017). However, the potential biological mechanisms are not understood. As donor sex affects the amount of circulating young RBCs in the blood product (Alshalani et al., 2019; Mykhailova et al., 2020), biological age of RBCs may play a role in adverse transfusion events.

During erythropoiesis, reticulocytes are released into the circulation where they mature completely to RBCs (Ney, 2011). Reticulocytes are characterized by their intracellular residual RNA (Lee et al., 1986; Schimenti et al., 1992) and the expression of the transferrin receptor (CD71) (Lebman et al., 1982; Liu et al., 2014). Both the RNA content and CD71 are gradually eliminated during maturation of RBCs (Koepke and Koepke, 1986). Changes in RBC structural properties include an increase in intracellular hemoglobin concentration and loss of surface area, leading to an increase in the density of cells (Mykhailova et al., 2020; Bizjak et al., 2015; Sparrow et al., 2006; D'Alessandro et al., 2013; Bosch et al., 1992; Lutz et al., 1992; Piomelli and Seaman, 1993).

Exposing the immune system to circulating immature RBCs, mainly reticulocytes, has been connected to some immunosuppressive effects in neonates and cancer patients (Elahi et al., 2013; Chan et al., 2014; Elahi, 2014; Liu et al., 2014; Namdar et al., 2017; Miller et al., 2018). This study hypothesized that the young RBC fraction contains more reticulocytes, which would suppress endothelial cell adhesion and activation, T cell proliferation, and cytokine secretion after whole blood stimulation.

## Materials and methods

### Young and old red blood cell preparation

Blood studies were approved by the Medical Ethical Committee of Sanquin Research and performed in accordance with the 2013 Declaration of Helsinki. Whole blood samples were collected from healthy volunteers. Plasma and buffy coat were discarded by centrifugation (1,500 xg, 10 min, 20°C) and RBCs were suspended in an equal volume of SAGM. Leukoreduction was done using Acrodisc WBC syringe filter (Pall Laboratory). Young RBCs and old RBCs were separated using a Percoll fractionation method as described previously (Mykhailova et al., 2020), with modifications. Briefly, 1.5 mL of each RBC samples was gently layered on 3 mL of different Percoll (GE Healthcare) densities (1.090, 1.093, 1.095, 1.098, 1.100, and 1.103 g/L) in 5 mL tubes (BD Falcon). Tubes were centrifuged (2,200 xg, 10 min, 20°C) with low acceleration and deceleration speeds (2 and 1 correspondingly), and the appropriate Percoll density was visually determined for each sample by choosing the lowest density that leads to a clear separation of young RBCs and old RBCs. Young RBCs and old RBCs were isolated from each sample by gently layering 3.5 mL of RBC on 10 mL of the corresponding appropriate Percoll density in 15 mL conical tubes. Tubes were centrifuged (2,200 xg, 15 min, 20°C) with low acceleration and deceleration speeds (2 and 1 correspondingly), and the top layer (young RBCs) was isolated from the bottom layer (old RBCs). Isolated cells were washed two times (at 2,200 xg, 10 min, 20°C) in SAGM to remove residual Percoll, suspended in an equal volume of SAGM, and stored at 4°C for further use.

### Reticulocyte quantification and quality assessment

Reticulocyte quantification was determined by percentage of CD71-positive cells, of thiazole orange (TO)-positive cells, and of reticulocytes. To determine the percentage of CD71-and TO-positive cells, 1 µL of RBCs (i.e., approximately 4 × 10^6^ RBCs) were incubated for 30 min at 4°C in the dark with a saturating concentration of an antibody mixture containing anti-CD71 (APC; BD Biosciences) and anti-CD235a (FITC; Biorad), Thiazole Orange (Sigma-Aldrich) and anti-CD235a (APC; Biorad), or IgG1 isotype negative control (FITC; ThermoFisher). Labeled cells were then washed twice, suspended in 200 µL of HEPES buffer, and acquired using the LSR Fortessa Flow Cytometer (BD Bioscience). 10,000 events were recorded for each sample. The analysis was performed with FlowJo v10 software (FlowJo, Ashland, OR). Reticulocyte percentage and RBC indices, including mean cell volume (MCV), mean cell Hb (MCH), mean cell Hb concentration (MCHC), and hemoglobin concentration (HGB), were analyzed by ADVIA 2120i Hematology System (Siemens).

### Endothelial cell culture

Human Lung Microvascular Endothelial Cells (HMVEC-L) were obtained from Lonza and cultured in EBM^TM^-2 Basal Medium supplemented with EGM^TM^-2 MV Microvascular Endothelial Cell Growth Medium SingleQuots^TM^ supplements (Lonza) in cell culture flasks (75 cm^2^, Greiner Cellstar) coated with 0.75% gelatine (BD Bioscience) at 37°C with 5% CO_2_, and passaged using trypsin/EDTA, trypsin neutralizing, and HEPES Buffered Saline solutions (Lonza). In this experiment, HMVEC-L cells at passage seven were used. After reaching 80%–90% confluence, cells were transferred to gelatin-coated 12 well cell culture plate (Greiner Cellstar).

For adhesion of RBCs to endothelial cells, RBCs were labeled with Vybrant™ DiD Cell-Labeling Solution (Thermo Fisher Scientific) by incubating 20 µL of RBCs and 20 µL of the labeling solution in 2 mL HEPES buffer for 20 min at 37°C. Labeled RBCs were then washed twice with HEPES buffer and suspended in 500 µL warm EBM medium. After that, 1 × 10^7^ labeled RBCs were added to each well and incubated for 5 h at 37°C with 5% CO_2_ in a humidified atmosphere. LPS (lipopolysaccharide)-stimulated wells were subjected to 50 ng/mL LPS (*Escherichia coli*, Sigma Aldrich) at the time of adding labeled RBCs to the confluent HMVEC-L cells. After 5 h of incubation, medium was aspirated from each well, spun down, and the supernatant was frozen for further testing. Wells were evenly washed three times with HEPES Buffered Saline solutions to remove unattached RBCs and briefly trypsinized as described earlier. After centrifugation (200 xg, 5 min, 4°C), cells were suspended in 1 mL HEPES buffer and acquired using the LSR Fortessa Flow Cytometer (BD Bioscience).

### Assessment of cell adhesion molecules

Human Magnetic Luminex Assay was used to measure the concentrations of E-selectin, intercellular adhesion molecule-1 (ICAM-1), syndecan-1, and von Willebrand Factor (vWF) in the HMVEC-L cell culture supernatants according to the manufacturer’s instructions (R&D Systems). Plates were read using a Bio-Plex 200 analyzer (Bio-Rad).

### T cell proliferation assessment

Peripheral blood mononuclear cells (PBMCs) were isolated from whole blood of healthy volunteers using Percoll (GE Healthcare) with a density of 1.076 g/ml. T cells were indirectly isolated from the PBMCs using Pan T cell isolation MACS (Miltenyi Biotec), in which all blood cells except T cells were depleted by magnetic separation. T cells were then labeled with carboxyfluorescein diacetate succinimidyl ester (CFSE) (Life Technologies). Subsequently, cells were washed and suspended in Iscove modified Dulbecco medium (IMDM; Gibco, Life Technologies) supplemented with fetal calf serum (Bodinco, Alkmaar, Netherlands), penicillin (Sigma-Aldrich), streptomycin (Sigma-Aldrich), glutamine (Sigma-Aldrich), and β-mercaptoethanol (Sigma-Aldrich). Cells were then cultured in 96 well flat-bottom plates (2 × 10^4^ T cells per well) for 5 days along with anti-CD3 (Sanquin, Amsterdam, Netherlands) and anti-CD28 (Sanquin) to induce proliferation. T cell proliferation was also assessed in the presence of neutrophils, as described previously (Aarts et al., 2019). Polymorphonuclear neutrophils (PMNs) were added (6 × 10^4^ cells per well) in the presence of tumor necrosis factor-alpha (TNFα; 10 ng/mL; PeproTech EC). Young RBCs and old RBCs were prepared from the same donor and were co-cultured (6 × 10^4^ cells per well) with T cells to assess the effect of RBC subpopulations on T cell proliferation. T cell proliferation was analyzed based on CFSE dilution using LSR Fortessa Flow Cytometer (BD Bioscience). 10,000 events were recorded for each sample. T cells were gated in P1 representing the original cells before stimulating cells for proliferation with anti-CD3 and anti-CD28 (Supplementary Figure S1A), and proliferated T cells were gated in P2 (Supplementary Figure S1B). The ratio of T cell proliferation was calculated by dividing the number of cells in P2 by the number of cells in P1.

### Whole blood stimulation assay

Whole blood (0.5 mL) from healthy volunteers was stimulated with 50 ng/mL LPS (*Escherichia coli*, Sigma-Aldrich) and incubated for 24 h in 0.5 mL RPMI 1640 Medium supplemented with L-glutamine (Gibco, ThermoFisher) in the presence or absence of either young RBCs or old RBCs derived from the same donor (5 × 10^7^ cells per well). After incubation, samples were centrifuged (600 xg, 10 min, 20°C), and supernatants were stored at −80°C until cytokines analyses. IL-6, IL-8, IL-10, and TNFα concentrations were measured using ELISA assay (R&D Systems).

### Statistical analysis

Two duplicates were run for each sample. The normality of the data was tested by the Shapiro-Wilk test and was visually inspected in Q-Q Plots. A Students t-test was performed when normality assumption was obtained; otherwise, a Mann-Whitney *U* test, to evaluate differences between two groups was performed. Comparisons between more than two groups were performed using a one-way ANOVA if data was normally distributed, otherwise a Kruskal-Wallis test, with correction for multiple comparisons. Unless stated otherwise, means and standard deviations (SD) were reported. A *p*-value of less than 0.05 was considered statistically significant. Statistical analyses were performed using SPSS® version 26.00 software. Graphical representation was generated using GraphPad Prism® version 8.3.0.

## Results

### Reticulocytes are mainly present in the young RBCs fraction

**FIGURE 1:**
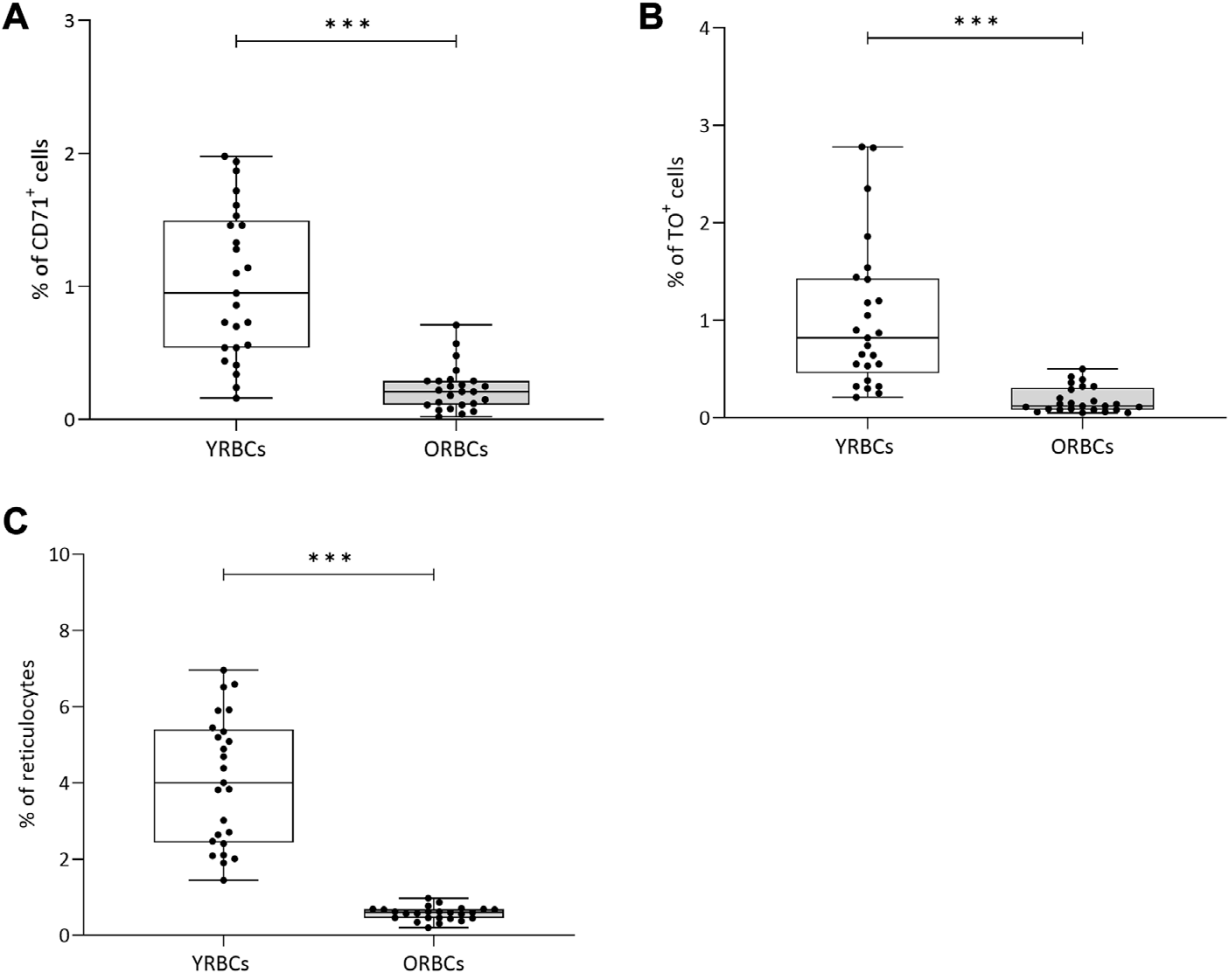
Percentage of reticulocytes after Percoll fractionation. Percentages of **(A)** CD71^+^ cells and **(B)** TO+ (Thiozole orange) cells were assessed using flow cytometry. **(C)** Percentage of reticulocytes cells was assessed using a hematology analyzer. YRBCs refers to young red blood cells, and ORBCs refer to old red blood cells. * denotes a significant difference between groups (*p < 0.05, **p < 0.01, ***p < 0.001). Blood samples were collected from healthy volunteers (*n* = 25).

Following Percoll fractionation, reticulocytes are more frequent in the young cell fraction compared to old cell fraction (Figure 1). Table 1 provides a summary of RBC indices. Young RBCs have a significantly lower HGB concentrations, MCH, and MCHC and higher MCV compared to old RBCs. No hemolysis was detected visually in the testing tubes.

**TABLE 1:** Red blood cells quality parameters after Percoll fractionation.

	YRBCs	ORBCs
MCV (fL)	99.08 ± 7.48	92.07 ± 4.73***
MCH (fmol)	1.78 ± 0.12	1.85 ± 0.12*
MCHC (mmol/L)	17.98 ± 1.14	20.11 ± 0.63***
HGB (mmol/L)	7.63 ± 1.07	9.13 ± 0.92***
RDW (%)	13.26 ± 0.73	13.19 ± 0.64

Data are shown as mean ± SD. YRBCs refers to young red blood cells, and ORBCs refer to old red blood cells. * denotes a significant difference between groups (*p < 0.05, **p < 0.01, ***p < 0.001). Blood samples were collected from healthy volunteers (*n* = 25).

### Incubation with young RBCs is associated with a reduction in the levels of endothelial activation markers compared to old RBCs

**FIGURE 2:**
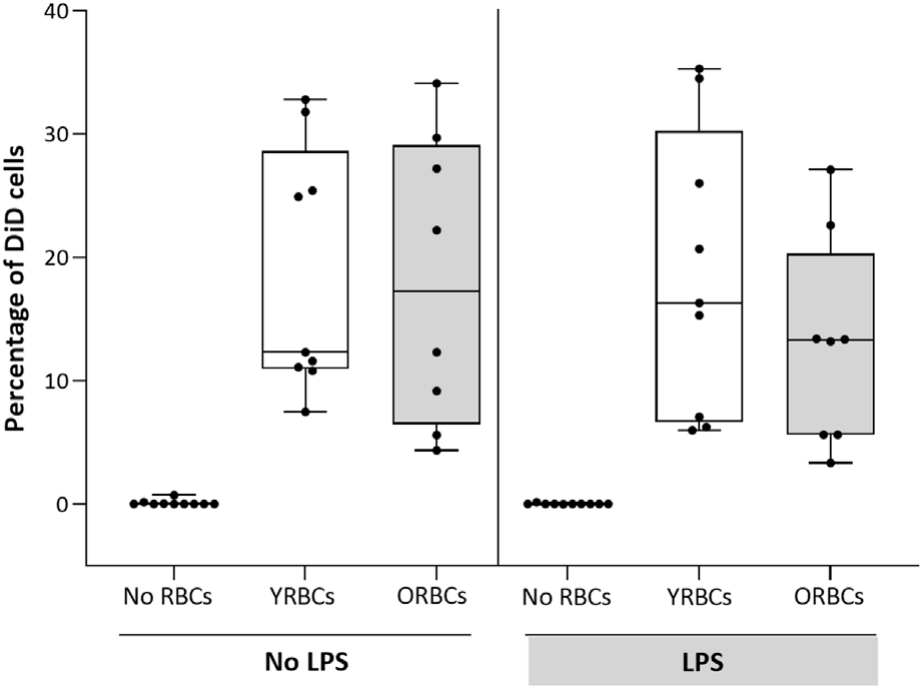
Adhesion of RBCs to endothelial cells. Young red blood cells (YRBCs) and old red blood cells (ORBCs) were stained with Vybrant™ DiD Cell-Labeling Solution and incubated with HMVEC-L for 5 h at 37 °C with 5% CO_2_ in presence and absence of LPS. *n* = 8–10 each.

**FIGURE 3:**
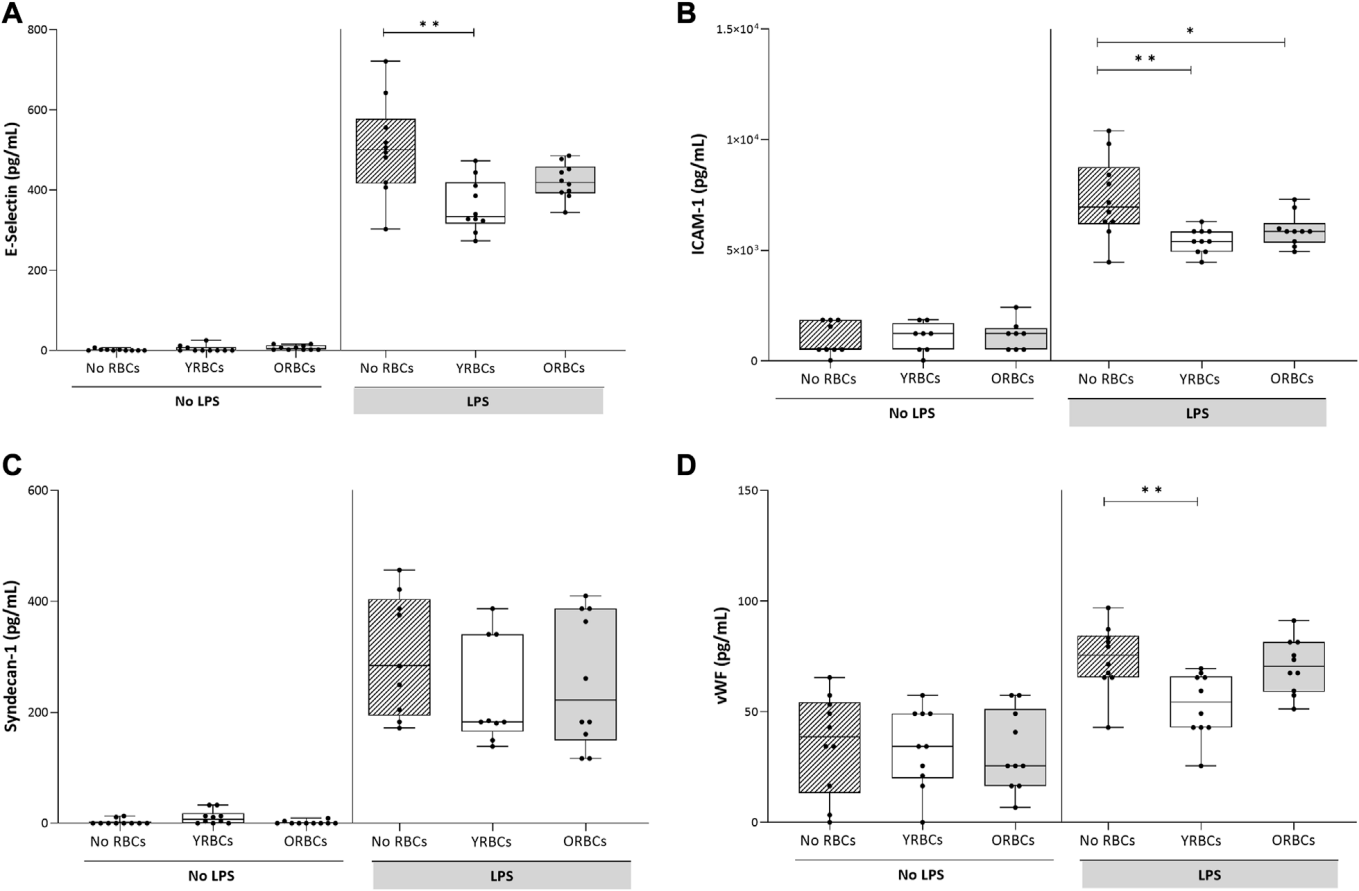
Endothelial activation markers after incubating young red blood cells (YRBCs) and old red blood cells (ORBCs) with endothelial cells. Concentrations of **(A)** E-selectin, **(B)** ICAM-1 **(C)** syndecan-1, and **(D)** Von Willebrand Factor were measured in presence and absence of LPS. * denotes a significant difference (*p-value < 0.05, ** *p-value* < 0.01, *** *p-value* < 0.001) when compared with no RBCs group. *n* = 9–10 each.

RBCs adhere to both activated and resting endothelial cells, but without differences between young RBCs or old RBCs (Figure 2). LPS stimulation resulted in increased secretion of endothelial activation markers, and co-incubation with RBCs did not further increase expression of these markers. On the contrary, the presence of young RBCs is associated with reduced level of E-selectin (360.05 pg/mL ± 65.69 pg/mL) compared to the condition without RBCs (504.89 pg/mL ± 118.86 pg/mL). Similarly, the concentration of vWF was significantly lower in the presence of young RBCs (53.07 pg/mL ± 14.57 pg/mL) compared to the condition without RBCs (74.12 pg/mL ± 15.05 pg/mL). Concentration of ICAM-1 was significantly lower in the presence of both young RBCs and old RBCs (5.4 × 10^3^ pg/mL ± 1.8 × 10^3^ pg/mL and 5.9 × 10^3^ pg/mL ± 0.73 × 10^3^ pg/mL; respectively) compared to the condition without RBCs (7.3 × 10^3^ pg/mL ± 1.8 × 10^3^ pg/mL). However, the significance level was higher in the presence of young RBCs compared old RBCs (*p-value* < 0.01 vs. *p*-value < 0.05) (Figure 3).

### Young RBCs do not differ from old RBCs in mediating T Cell proliferation or cytokine release following whole blood stimulation

**FIGURE 4:**
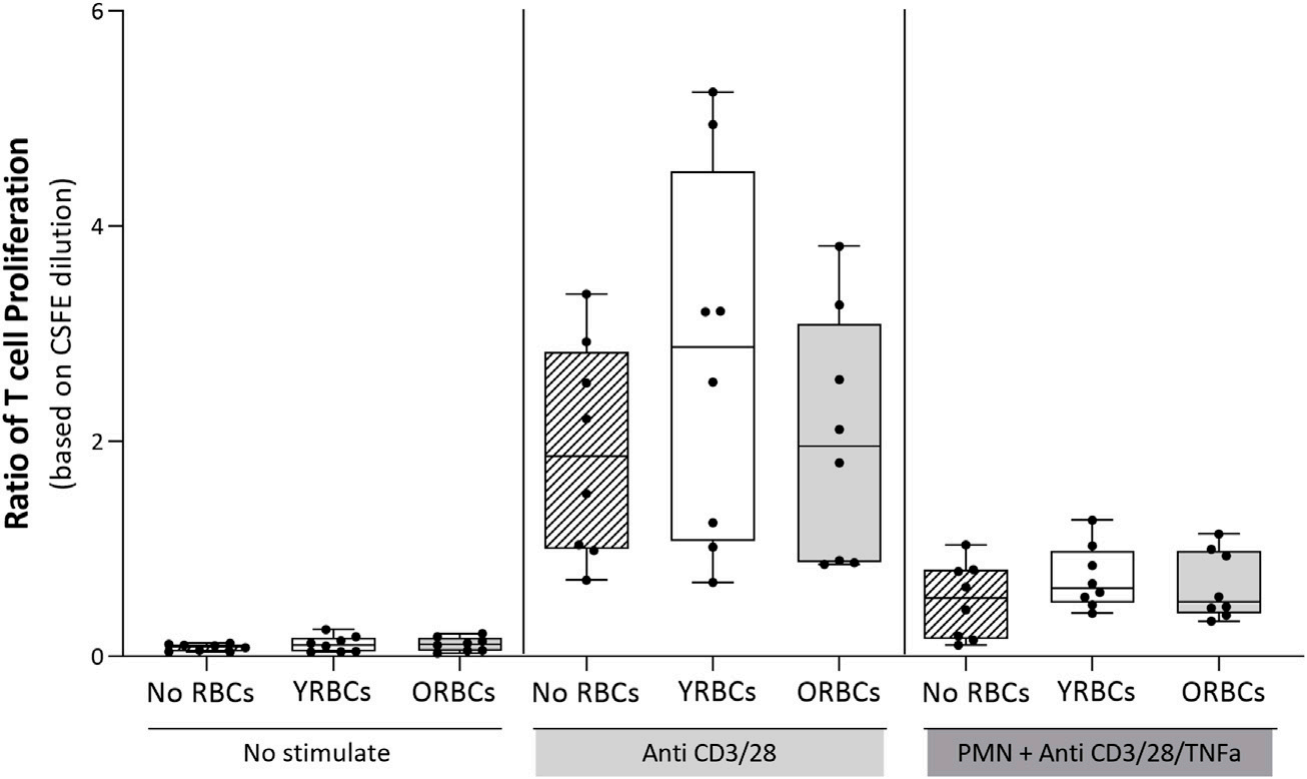
The ratio of T cell proliferation. T cells were incubated with young red blood cells (YRBCs) and old red blood cells (ORBCs) in the presence or absence of the stimulus (anti-CD3 and anti-CD28). T cell proliferation was suppressed with polymorphonuclear neutrophils (PMNs) in the presence of anti-CD3, anti-CD28, and TNFα. *n* = 8 each.

**FIGURE 5:**
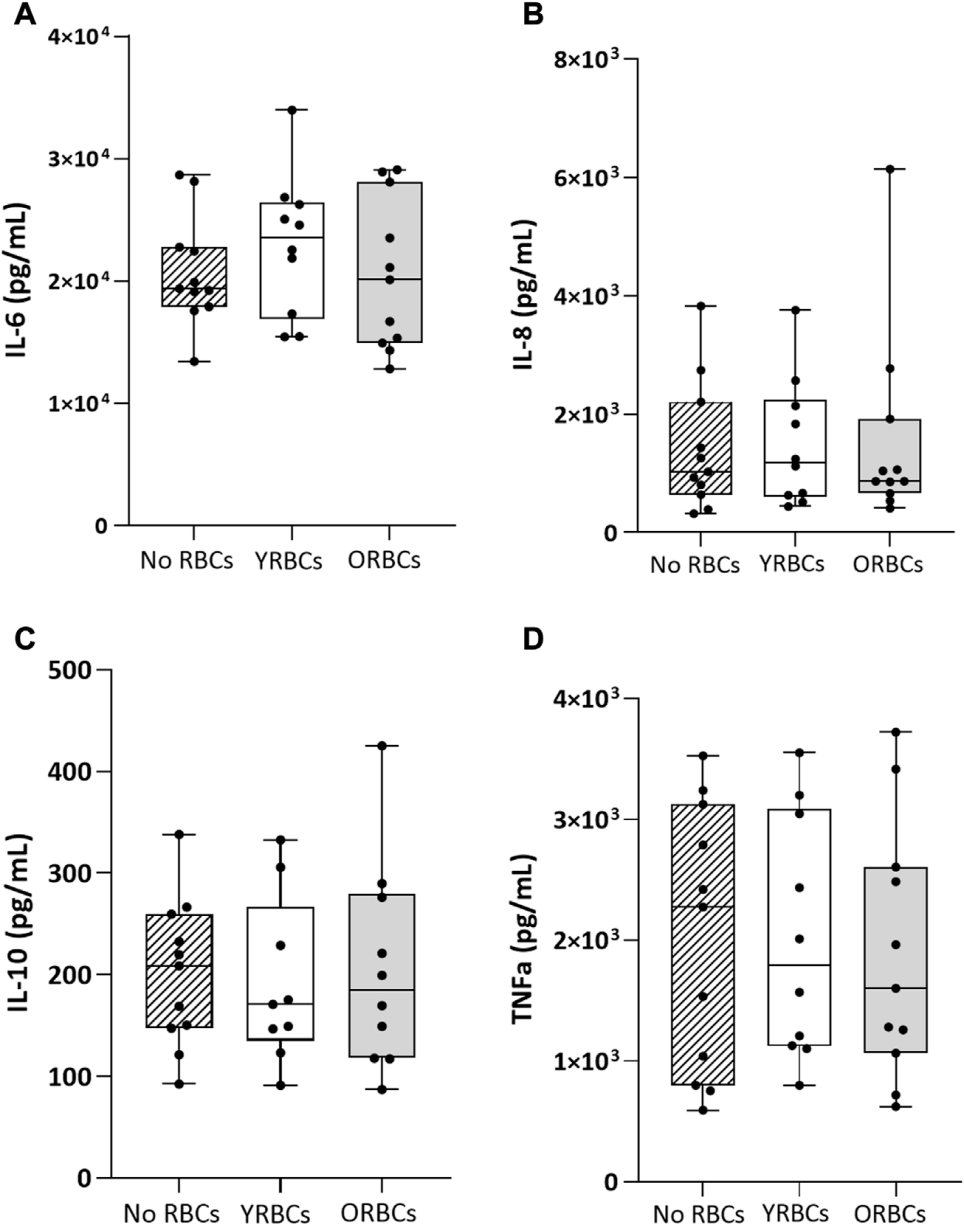
Cytokine levels after whole blood stimulation in presence or absence of young red blood cells (YRBCs) or old red blood cells (ORBCs). Concentrations of **(A)** IL-6, **(B)** IL-8 **(C)** IL-10, and **(D)** TNFα were measured after stimulating whole blood with LPS. *n* = 9–11 each.

Figure 4 shows the ratio of T cell proliferation in the presence of young RBCs or old RBCs. Overall, no significant difference between groups were evident in the presence or absence of the stimulus. Young RBCs increased the ratio of T cell proliferation to 2.76 ± 1.73, while it remained 1.62 ± 0.73 in the condition with no RBCs and 2.02 ± 1.14 in the presence of old RBCs, although not reaching statistical significance (*p-value* = 0.19). In addition, the presence or absence of RBCs did not affect cytokine responses after whole blood stimulation with LPS (Figure 5).

## Discussion

The current study shows that the Percoll fractionation concentrated reticulocytes in young RBCs fraction, as found before (van Cromvoirt et al., 2021). We further show that the presence of young RBCs associated with lower levels of endothelial activation markers compared to old RBCs, while not altering T cell proliferation or cytokine secretion following whole blood stimulation.

It is known that transfusing young RBCs yields a higher survival in recipients’ circulation compared to unfractionated (standard) RBCs (Piomelli et al., 1978; Graziano et al., 1982; Triadou et al., 1986), although clinical data available to date on cost-effectiveness of transfusing young RBCs may not be favorable, giving the expense, time, and work required to produce young RBC product in comparison to standard RBC product (Cohen et al., 1984; Marcus et al., 1985; Pisciotto et al., 1986). In addition, transfusion of young RBCs increases the time intervals between transfusions compared to standard RBCs (Triadou et al., 1986; Montoya, 1993; Sharma et al., 2008). Furthermore, during storage, young RBCs have lower hemolysis, rigidity, and oxidized HGB levels compared to old RBCs (Antonelou et al., 2012; Mykhailova et al., 2020). This study reported a higher reticulocyte percentage in young RBCs fraction compared to old RBCs. This finding further supports the idea that red cell density is highly related to the age of RBCs (Piomelli and Seaman, 1993). Of note, the percentage of reticulocytes was higher in the hematology analyzer compared to the flow cytometry assay (CD71 and TO). A possible explanation is that the hematology analyzer classifies cells according to their structural and morphological features based on the absorbance of Oxazine 750, a nucleic acid dye that stains the intracellular RNA (Harris et al., 2005). However, CD71 and TO do not account for the cellular characteristics of reticulocytes, and they are more specific for RBCs expressing CD71 on their surfaces or having intracellular RNA, respectively. In either way, the difference between young RBCs and old RBCs was consistent in all reticulocyte expression assays.

The current study shows that less dense young RBCs, but not old RBCs, associated with lower level of endothelial activation markers. An explanation for the inhibitory effect of young red cells may be related to the presence of adhesion receptors. While mature RBCs circulate fluently in blood vessels without adhering to vascular endothelial cells, less mature erythroid cells express cell adhesion molecules which are eliminated before turning to mature RBCs (Spring and Parsons, 2000; Telen, 2000). It is therefore possible that those markers are not entirely lost from the membrane of reticulocytes, which then interact with the endothelium. Also, in line with our findings, two recent studies have shown that enriching CD71 positive erythroid cells was associated with immunosuppressive effects (Dunsmore et al., 2017; Namdar et al., 2017), whereas depletion of CD71 positive erythroid cells restored resistance of immune cells against bacterial growth (Elahi et al., 2013; Sano et al., 2021). A note of caution should be mentioned here when comparing these previous findings with our findings since previous study involve animal and cord blood subjects with abundancy of CD71 positive cells. The actual mechanism of how CD71 positive cells affect the host immune response is not clear. One possibility is that erythroid cells co-express arginase-2, an enzymatic activity involved in inhibiting cytokine production (Delyea et al., 2018). Furthermore, another recent study has shown that CD71 positive erythroid cells express inhibitory receptors, such as V-domain Immunoglobulin Suppressor of T Cell Activation (VISTA), which have a pivotal role in suppressing T cell activation through regulatory cytokines (Shahbaz et al., 2018). Whether these inhibitory markers are present in early erythroid precursors expressing CD45 and CD71 or in later erythroid cells (CD45^−^ CD71^+^) and/or in adult or neonates is controversial (Elahi et al., 2013; Zhao et al., 2018).

Of note, the age of RBCs did not impact T cell proliferation or cytokine expression following stimulation with LPS. Possibly, RBCs were incubated with T cells and whole blood from the same donor while the endothelial cells were purchased from an external company. Thereby, whether incubating young RBCs with blood of an ABO-matched recipient will modulate host the immune response remains to be determined.

This study is limited in several ways. First, the small sample size may decrease the power to detect differences between groups. Second, the current study did not account for donor sex differences for the functional characteristics of RBC subpopulations (Mykhailova et al., 2020). There could be unaccounted differences related to donor sex. Third, the endothelial flow model is more relevant to the nature of RBCs than a static model. Fourth, the use of *in vitro* system with immortalized endothelial cell lines, and, therefore, translating findings of this study to human is questionable. Fifth, we did not stain for reticulocytes so we cannot guarantee that young non-reticulocyte cells may have contributed to results. Also, very old cells may swell and hence be part of the less dense fraction. Furthermore, this study used SAGM as a preservative solution for RBCs which may influence cellular volume due to its hypertonic condition, which has been investigated previously (Zehnder et al., 2008). Last, the direct mechanisms of RBC-endothelial adhesion and activation were not investigated in this study.

In conclusion, the current study assessed the potential immunomodulatory effects of the age of RBCs. Young RBCs contain more reticulocytes than old RBCs and are associated with reduced level of endothelial activation markers compared to old RBCs. Young RBCs did not have an impact on T cell proliferation or cytokine responses in a whole blood stimulation assay. Despite its exploratory nature, this current study offers age of RBCs as a potential novel mechanism of transfusion associated adverse outcomes.

## Data Availability

The raw data supporting the conclusion of this article will be made available by the authors, without undue reservation.
