# Supplementary material for: The impact of biological age of red blood cell on in vitro endothelial activation markers
Source: Front Physiol. 2023 Mar 8;14:1127103. doi: 10.3389/fphys.2023.1127103 (PMC10030615; doi:10.3389/fphys.2023.1127103)
Supplement: Supplementary file 1 [file Table1.DOCX]

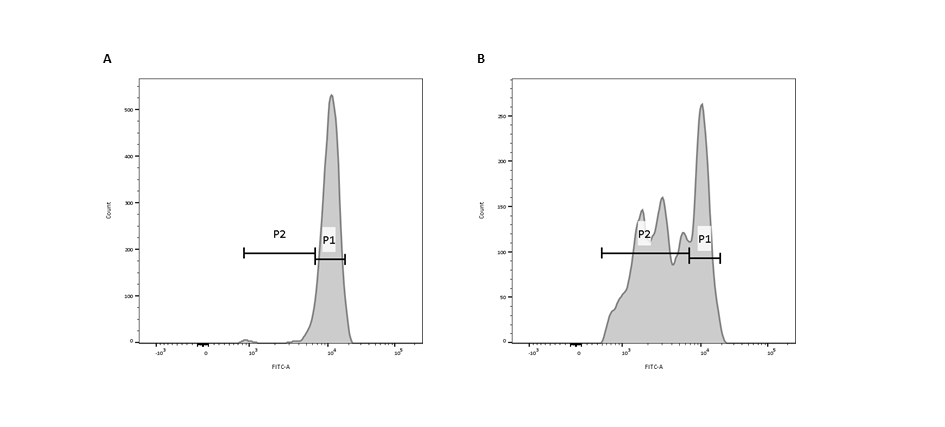


Supplemental Figure 1 (A) T cells were gated in P1 representing the original T cells before stimulating cells for proliferation with Anti-CD3 and anti-CD28, and (B) proliferated T cells were gated in P2. The ratio of T cell proliferation was calculated by dividing the number of cells in P2 by the number of cells in P1.
